# FLIPPING THE SCRIPT: WHY DO STUDENTS CHOOSE GERONTOLOGY AND GERIATRICS?

**DOI:** 10.1093/geroni/igae098.1038

**Published:** 2024-12-31

**Authors:** Isaac Asirifi Boateng, Mark Staley, Jess VanderWerf

**Affiliations:** Saint Cloud State University, Saint Cloud, Minnesota, United States; University of Nebraska Lincoln, Lincoln, Nebraska, United States; University of South Florida, Tampa, Florida, United States

## Abstract

Much of the literature on workforce shortages and low enrollments in gerontology and geriatrics education focuses on the barriers - essentially “why don’t students choose to go into gerontology”? This presentation reframes the question to instead consider why gerontology and geriatrics students DO choose the field? Three student perspectives - an undergraduate pre-med certificate student, a master’s student in Gerontology, and a doctoral student in aging studies offer insight into the factors drawing them to gerontology and geriatrics. In addition to sharing their personal journeys, they also share what they have learned from their gerontology classmates and peers at institutions across the United States.

